# Radiological and Clinical Value of 7T MRI for Evaluating 3T-Visible Lesions in Pharmacoresistant Focal Epilepsies

**DOI:** 10.3389/fneur.2021.591586

**Published:** 2021-03-02

**Authors:** Z. Irene Wang, Se-Hong Oh, Mark Lowe, Mykol Larvie, Paul Ruggieri, Virginia Hill, Volodymyr Statsevych, Doksu Moon, Jonathan Lee, Todd Emch, James Bena, Ingmar Blümcke, William Bingaman, Jorge A. Gonzalez-Martinez, Imad Najm, Stephen E. Jones

**Affiliations:** ^1^Epilepsy Center, Cleveland Clinic, Cleveland, OH, United States; ^2^Division of Biomedical Engineering, Hankuk University of Foreign Studies, Yongin, South Korea; ^3^Imaging Institute, Cleveland Clinic, Cleveland, OH, United States; ^4^Department of Radiology, Feinberg School of Medicine, Northwestern University, Chicago, IL, United States; ^5^Department of Quantitative Health Science, Cleveland Clinic, Cleveland, OH, United States; ^6^Institute of Neuropathology, University Hospitals Erlangen, Erlangen, Germany; ^7^Department of Neurosurgery, Cleveland Clinic, Cleveland, OH, United States

**Keywords:** epilepsy, MRI, 7T, ultra-high field, presurgical evaluation, imaging, seizures

## Abstract

**Objective:** The recent FDA approval of the first 7T MRI scanner for clinical diagnostic use in October 2017 will likely increase the utilization of 7T for epilepsy presurgical evaluation. This study aims at accessing the radiological and clinical value of 7T in patients with pharmacoresistant focal epilepsy and 3T-visible lesions.

**Methods:** Patients with pharmacoresistant focal epilepsy were included if they had a lesion on pre-operative standard-of-care 3T MRI and also a 7T research MRI. An epilepsy protocol was used for the acquisition of the 7T MRI. Prospective visual analysis of 7T MRI was performed by an experienced board-certified neuroradiologist and communicated to the patient management team. The clinical significance of the additional 7T findings was assessed by intracranial EEG (ICEEG) ictal onset, surgical resection, post-operative seizure outcome and histopathology. A subset of lesions were demarked with arrows for subsequent, retrospective comparison between 3T and 7T by 7 neuroradiologists using a set of quantitative scales: lesion presence, conspicuity, boundary, gray-white tissue contrast, artifacts, and the most helpful sequence for diagnosis. Conger's kappa for multiple raters was performed for chance-adjusted agreement statistics.

**Results:** A total of 47 patients were included, with the main pathology types of focal cortical dysplasia (FCD), hippocampal sclerosis, periventricular nodular heterotopia (PVNH), tumor and polymicrogyria (PMG). 7T detected additional smaller lesions in 19% (9/47) of patients, who had extensive abnormalities such as PMG and PVNH; however, these additional findings were not necessarily epileptogenic. 3T−7T comparison by the neuroradiologist team showed that lesion conspicuity and lesion boundary were significantly better at 7T (*p* < 0.001), particularly for FCD, PVNH and PMG. Chance-adjusted agreement was within the fair range for lesion presence, conspicuity and boundary. Gray-white contrast was significantly improved at 7T (*p* < 0.001). Significantly more artifacts were encountered at 7T (*p* < 0.001).

**Significance:** For patients with 3T-visible lesions, 7T MRI may better elucidate the extent of multifocal abnormalities such as PVNH and PMG, providing potential targets to improve ICEEG implantation. Patients with FCD, PVNH and PMG would likely benefit the most from 7T due to improved lesion conspicuity and boundary. Pathologies in the antero–inferior temporal regions likely benefit less due to artifacts.

## Introduction

MRI plays an important role for the presurgical evaluation of patients with pharmacoresistant focal epilepsy. MRI's ability to detect potential epileptogenic lesions has markedly improved in the past decades, largely due to improved signal-to-noise ratio (SNR) resulting from increasing magnetic field strength (0.5T→ 1.5T→ 3T). After successfully entering the clinical practice in 2002 upon FDA approval, 3T MRI quickly became a mainstay of clinical imaging when high spatial resolution and contrast-to-noise ratio are required, particularly for patients with epilepsy. Analogously, the recent FDA approval of the first 7T MRI scanner for clinical diagnostic use in October 2017 will likely increase the usage of 7T for epilepsy. There have been a number of studies on structural 7T MRI in patients with pharmacoresistant focal epilepsy undergoing presurgical evaluation. These studies provided initial evidence that the improved signal-to-noise ratio from 7T MRI can lead to better detection/depiction of some epileptic lesions, such as focal cortical dysplasia (FCD), polymicrogyria (PMG), and vascular malformations (1–10).

Since the installation of a Siemens Magnetom 7T at our institution in 2014, we have been performing a prospective study on more than 100 pharmacoresistant focal epilepsy patients evaluated for epilepsy surgery. This study reports the findings on the subset of these patients (*N* = 47) who already had positive 3T MRI [the nonlesional 3T patients were published recently in a separate study (10)]. Of note, we report in the current study the additional 7T findings in relationship to the ICEEG, surgery, and seizure outcome results. We hypothesize that the improved spatial resolution, signal-to-noise ratio, and contrast-to-noise ratio brought by 7T MRI will improve delineation of epileptic lesions compared to 3T MRI.

The second part of the current study was designed to address the issue that prior 7T studies reached agreement in image interpretation through consensus-based discussions from a small number of neuroradiologists or epileptologists, and the actual agreement data were not available. Here, we included a team of seven clinical neuroradiologists, who used a quantified scoring system to compare a set of features for lesions identified on both 7T and 3T MRI scans. We hypothesize the neuroradiologist team review data would show a subset of lesions benefiting significantly from 7T in terms of lesion conspicuity and boundary.

## Patients and Methods

### Study Design and Patient Selection

This is a single-center study on patients with pharmacoresistant focal epilepsy evaluated for resective surgery at the Cleveland Clinic Epilepsy Center from 2014 to 2018. Since the MRI used for this study (Siemens Magnetom) predated the FDA-approved Siemens Terra, all scans were performed as a research study approved by the Cleveland Clinic institutional review board (IRB). Patients were included if they had a positive 3T MRI based on official radiology report, with lesion findings that may be relevant to the epilepsy. Patients were excluded if they: (1) could not lay still in the scanner, which included claustrophobia, psychiatric conditions, cooperation issues, and low body weight (<30 kg); (2) had metal objects in their body/head incompatible with 7T MRI; (3) unable to provide informed consent or assent to study; or (4) were pregnant. After acquiring the 7T MRI, the scan was prospectively reviewed in comparison with lower field imaging by a board-certified neuroradiologist with 13 years of expertise in epilepsy imaging (SEJ), for the immediate purpose of confirmation of the lower-field lesion. Any additional findings at 7T that might be of clinical value were communicated with the patient's epileptologist. The strategies for intracranial EEG (ICEEG) and surgical resection were discussed during a patient management conference (PMC). Discussions were based on multimodal data from scalp EEG video monitoring, 3T MRI, FDG-PET, subtraction ictal SPECT co-registered to MRI (SISCOM), and Magnetic Source Imaging (MSI). If 7T MRI showed additional findings, these results are shown at the PMC as well. Should ICEEG be necessary, additional PMCs were held to discuss the invasive evaluation findings and the final strategy for surgical resection/ablation.

### MRI Acquisition Parameters

We used a standard epilepsy protocol on a 7T MRI scanner (Magnetom, Siemens, Erlangen, Germany) with a head-only circularly polarized transmit and 32-channel phased array receive coil (Nova Medical, Wilmington, MA), consistent with our previous study (3). Sequence parameters were as follows: *3D T1-MP2RAGE:* sagittal acquisition, TR/TE = 6,000/3 ms, TI1/TI2 = 700/2,700 ms, flip angle 1/flip angle 2 = 4/5°, 0.75 mm isotropic-voxel resolution, 208 slices, total acquisition time (TA) = 9 min 32 s; 2D T2*-GRE (Spoiled-Gradient Echo): axial and oblique coronal acquisition, TR/TE = 2,290/17.8 ms, flip angle = 23°, in-plane resolution = 0.38 × 0.38 mm^2^, slice thickness = 1.5 mm, 60 slices, no gap, TA = 9 min 50 s; *2D FLAIR*: axial and oblique coronal acquisition, TR/TE = 9,000/124 ms, TI = 2,600 ms, in-plane resolution = 0.75 × 0.75 mm^2^, slice thickness = 2 mm, 45 slices, 30 % gap, TA = 3 min 2 s; *3D SWI* (susceptibility weighted imaging, included only for selected cases such as vascular malformations): TR/TE = 23/15 ms, flip angle = 20°, voxel size = 0.49 × 0.49 × 0.8 mm^3^, 144 slices, TA = 8 min 16 s. Two dielectric calcium titanate pads with passive B1 shimming were used to improve the signal loss in the temporal lobes. The MRI scans obtained as standard care for the epilepsy patients were performed on a 3T scanner (Skyra, Siemens, Erlangen, Germany) using a 32-channel phased array receive coil (Nova Medical, Wilmington, MA) with similar physical characteristics as the one used for 7T. Sequence parameters used for the 3T epilepsy protocol were: 3D T1-MPRAGE: coronal acquisition, 0.8 × 0.8 × 1 mm voxel resolution, 256 slices, TR/TE = 1,800/2.56 ms, TI = 900 ms, flip angle = 10°, TA = 3 min 50 s; 2D T2-TSE (Turbo Spin Echo): oblique coronal acquisition, in-plane resolution = 0.56 × 0.56 mm, slice thickness = 3.0 mm, 49 slices, 20% gap, TR/TE = 4,000/78 ms, flip angle = 150°, TA = 3 min 22 s; 2D FLAIR: axial or oblique coronal acquisition, in-plane resolution = 0.7 × 0.7 mm, slice thickness = 3 mm, 49 slices, 20% gap, TR/TE = 8,640/128 ms (axial) or 8,100/81 ms (coronal), TI = 2,500/2,380 ms, flip angle = 150°, TA = 4 min 21 s/4 min 5 s; 2D SWI: axial acquisition, in-plane resolution 0.9 × 0.9 mm, slice thickness = 2.5 mm, 60 slices, 0% gap, TR/TE = 27/20 ms, flip angle = 15°, TA = 2 min 40 s (included only for selected cases such as vascular abnormalities).

### Concordance Between 7T Findings, ICEEG, and Surgery Location

The 7T images were coregistered with the CT obtained immediately after ICEEG implantation and the postoperative MRI using Curry 7 (Compumedics Neuroscan, Hamburg, Germany). The ICEEG contacts were marked based on the center of highest intensity on the CT images, and overlaid on the MRI. We obtained the ICEEG ictal onset from a review of the clinical report which was finalized based on PMC consensus. For assessing the relationship between lesion location and the ICCEG ictal onset, we used the same concordance scheme as previously published (11). Briefly, if the location of the 7T-detected lesion matched the ICEEG ictal onset zone, they were considered *concordant* (7T = ICEEG). If the 7T-detected lesion included the ICEEG ictal onset zone but occupied a bigger brain area, they were considered *concordant-plus* (7T > ICEEG). Conversely, if the ICEEG ictal onset zone included the 7T-detected lesion but occupied a bigger brain area, they were considered *concordant-minus* (7T < ICEEG). If there was only partial overlap between the 7T-detected lesion and ICEEG ictal onset zone, they were considered *partially concordant*. If there was no overlap between the 7T-detected lesion and ICEEG ictal onset zone, they were considered *discordant*. For assessing whether the lesions had been completely included in resection, considering post-operative movement of tissue around the resection cavity, as well as geometric distortion in MRI and image coregistration error, the lesions were considered as inside resection if the borders were completely within or <5 mm outside of the resection margin.

### Pathology, Surgery, and Seizure Outcomes

Surgical specimens were reviewed by an experienced neuropathologists (IB). FCD and hippocampal sclerosis (HS) were classified according to previously published ILAE classifications (12, 13). Tumor classification followed the new edition of the WHO classification scheme from 2016 (14). Post-operative seizure outcomes were classified according to Engel's classification system (15).

### Retrospective Review by Neuroradiologist Team

In a subset of patients, retrospective review by neuroradiologist team was performed on paired 7T and 3T studies. An experienced neuroradiologist SEJ performed an initial review of the 7T and 3T scans, identifying the lesions in each study with an overlaid arrow and saving it as a “Key image” into the picture archiving and communication system (AGFA IMPAX 6, Mortsel, Belgium). Guided by the arrows, the demarked lesions in both the 7T and 3T studies for each patient were subsequently reviewed by a group of seven neuroradiologists (other than SEJ). Note that the purpose of the team review was not the discovery and identification of an unknown lesion (which would be a considerably larger study), but a comparative assessment of imaging characteristics of a given lesion, hence the role of the arrows. This assessment evaluated the following imaging features for each lesion:

1. Presence of the lesion (0 [absent], 1 [equivocal], and 2 [present]), independently assessed for each set of 7T and 3T studies;
2. Lesion conspicuity (−2 [3T definitely better], −1 [3T mildly better], 0 [equal], 1 [7T mildly better], and 2 [7T definitely better]);
3. Lesion boundary (0 [not clearly visible], 1 [clearly visible]);
4. Comparison of gray-white tissue contrast on MPRAGE (−2 [3T definitely better], −1 [3T mildly better], 0 [equal], 1 [7T mildly better], and 2 [7T definitely better]);
5. Presence of artifacts affecting lesion conspicuity, such as motion, pulsation, and inhomogeneity;
6. Most helpful sequence for diagnosis, independently assessed for both 7T and 3T.

The reviewers were lastly asked to provide their diagnosis of the lesion based on 3T and 7T separately. While the key image was only provided to guide the reviewer to the location of the lesion to be evaluated, to more closely approximate clinical practice, the reviewers were allowed to evaluate the lesions using all sequences with their routine clinical DICOM viewer (AGFA IMPAX 6). The evaluation was solely based on imaging features alone, and reviewers were not given any clinical information, surgical site or other imaging information. While the reviewers were not explicitly given any information on whether the images were from a 3T or 7T study, identification between the two field strengths was not difficult for experienced neuroradiologists. The patient scans were intermixed with 3 normal control scans (two females, one male, age = 30, 27, and 34, respectively), where the arrows pointed to normal cortical regions on the “Key Images,” so that the reviewers were not obligated to report a lesion. The percentage or number of intermixed normal control scans was not known to the reviewers. If multiple pre-operative 3T studies existed, the one with the best image quality was selected for review. If a patient had multiple lesions, each lesion was reviewed separately.

### Statistical Analyses

Statistical analyses were performed using SAS software (version 9.4; Cary, NC). Agreement analyses among the seven neuroradiologists were performed using R (version 3.3; Vienna, Austria). Raw agreement across all raters was calculated, and then Conger's kappa for multiple raters was performed which provided chance-adjusted agreement statistics. Values of kappa between 0.0 and 0.2, 0.2 and 0.4, 0.4 and 0.6, 0.6 and 0.8, and 0.8 and 1.0 were considered poor, fair, moderate, good, and excellent, respectively. Mean levels for various outcomes were estimated using linear mixed effect models. Predicted probabilities for binary outcomes were estimated using a logistic regression with generalized estimating equations, and comparisons were performed. Comparisons of 3T and 7T on ordered outcomes were performed using proportional odds logistic regression with generalized estimating equations. Subgroup analyses on agreement tests were performed for five major pathology types: FCD, HS, tumor, PMG, and PVNH.

## Results

### Overall Cohort Overview (*N* = 47)

A total of 47 patients were included in the first part of the study for prospective review (23 males/24 females, 39 right-handed/7 left-handed/1 ambidextrous). The average age was 30.5 years (median = 26, range = 14–64). The average epilepsy duration was 14.5 years (median = 13, range = 1–50).

Radiological diagnosis based on the 3T MRI included 7 with unilateral HS, 1 with bilateral hippocampal signal increase suspicious for HS, 1 with unilateral hippocampal signal/volume increase of uncertain etiology, nine with FCD, one with FCD and HS, five with low-grade neoplasm, one with hippocampal cysts, two with cavernous malformation, two with DVA, one with DVA and FCD, seven with PVNH, five with PMG, four with chronic infarct, and one with tuberous sclerosis complex.

Of the 47 patients in the overall cohort, 20 patients (20/47, 43%) underwent additional ICEEG (18 SEEG and 2 subdural grids with depth electrodes). A total of 34 patients (34/47, 72%) underwent subsequent surgery (28 resective, 6 laser ablation). Complete seizure freedom (no auras) was achieved in 25 patients at 1-year follow up (25/34, 74%). Histopathology was reviewed in the 28 patients who underwent resective surgery with sufficient tissue; findings included four HS type I, one HS type II, four FCD type IIb, three FCD type IIa, one FCD IIIa, one FCD IIId, two PVNH, one cavernous malformation, one DNET grade I, one ganglioglioma grade I, 1 HS type I + FCD IIb (dual pathology), 1 HS type II + glial scars (dual pathology), 1 venous angioma + mMCD II (double pathology), one MCD with tubers, and five negative examinations.

### Clinical Yield of 7T on the Overall Cohort

**Figure 1:**
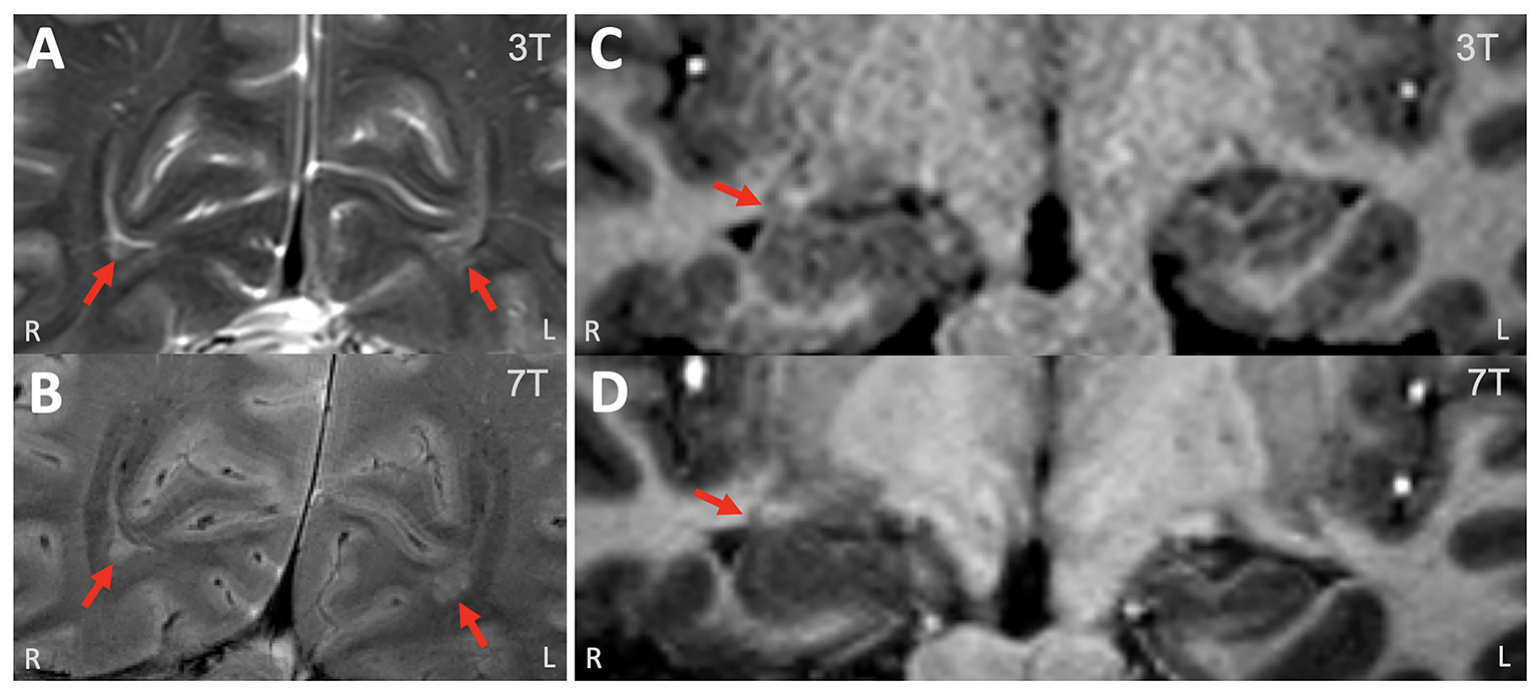
Example of a patient who had additional peri-ventricular nodular heterotopia lesions revealed on 7T. **(A)** 3T T2w TSE (coronal, zoomed in); **(B)** 7T T2*w GRE (coronal, zoomed in). These bi-occipital nodules (arrows) were previously overlooked on 3T. **(C)** 3T T1w MPRAGE (coronal, zoomed in); **(D)** 7T T1w MP2RAGE (coronal, zoomed in). Review of the 7T showed the enlargement of the right hippocampus head/amygdala was due to infiltration of the nodules, while the 3T finding was suspicious for underlying low-grade neoplasm vs. hamartoma. ICEEG showed ictal onset in the right hippocampus and amygdala, right entorhinal cortex and right temporal pole. A right temporal lobectomy including mesial structures was performed. The patient was seizure-free for 18 months but had seizures thereafter (with reduced frequency). Pathology showed nodular heterotopia.

**Figure 2:**
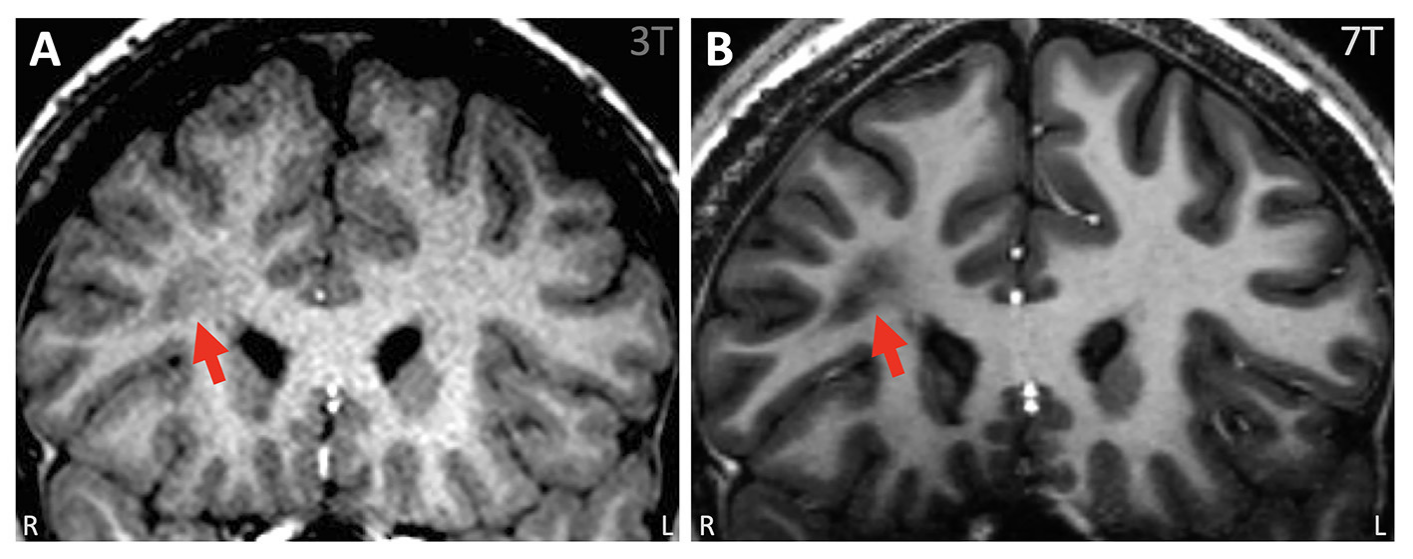
Example of ambiguous white matter lesion, the nature of which was further clarified by 7T. **(A)** 3T T1w MPRAGE (coronal) showing an unusual lesion in the right frontal lobe suggestive of a transmantle FCD. It was unclear if the lesion was related to his epilepsy because all his other tests pointed posteriorly to this region. **(B)** 7T T1w MP2RAGE (coronal). With higher detail due to smaller voxels from a 7T, it was verified that the lesion has no frank connection to the cortex, the adjacent cortex was normal with no gray-white blurring, and the abnormality was most likely not FCD but nonspecific ischemic change.

Table 1 summarizes the variety of pathology per 3T MRI and the corresponding clinical yield of 7T in each category. A total of 13 patients had additional findings detected on 7T (13/47, 28%). There were two patients whose 7T could not re-demonstrate the 3T lesions (2/47, 4%). The main yield of additional information was seen in the patients who had already extensive or multifocal abnormalities such as PMG, PVNH (example in Figure 1), perinatal infarct and TSC; for these patients, additional smaller abnormalities were frequently seen on the ipsilateral or the contralateral side (19%, 9/47). 7T detected a previously unseen FCD lesion in one patient who had HS as the pathology noted on 3T MRI (1/47, 2%), which was confirmed by subsequent ICEEG ictal onset and seizure freedom. The additional anatomical detail brought by 7T was useful in clarifying the nature of the lesion in one patient with likely non-specific ischemic change (Figure 2). In two patients, the lesions on 3T report could not be re-demonstrated on 7T (one unilateral HS became negative, one parieto-occipital FCD became negative) due to artifacts and partial volume effects at 3T, respectively; ICEEG/surgery performed subsequently were consistent with the 7T impression (not 3T).

**Table 1:** The clinical yield of 7T in the overall cohort of 47 patients, tabulated by the radiological diagnosis based on 3T MRI.

**Number of pts**	**Radiological diagnosis per 3T MRI**	**Number of pts with additional finding per 7T review**	**Description of additional finding per 7T review**
7	Unilateral HS	2	1/7: additional FCD seen
			1/7: HS not re-demonstrated
1	Bilateral hippocampal signal increase, right > left	1	HS confirmed to be unilateral (right-sided)
1	Unilateral hippocampal signal/volume increase	0	N/A
9	FCD	1	1/9: FCD not re-demonstrated
1	FCD+HS	0	N/A
5	Tumors	0	N/A
1	Cysts	1	Additional contralateral cysts seen
2	Cav Mal	0	N/A
2	DVA	1	1/2: additional contralateral microbleed was seen.
1	DVA+FCD	0	N/A
7	PVNH	2	1/7: additional ipsilateral PVNH seen
			1/7: additional ipsilateral PVNH+PMG seen
5	PMG	4	3/5: additional contralateral PMG seen
			1/5: additional ipsilateral PVNH seen
4	Chronic infarct	2	1/4: additional ipsilateral chronic infarct seen
			1/4: nature of lesion better clarified (3T: suspicious of malformation of cortical development; 7T: no connection to cortex, most likely nonspecific ischemic change)
1	TSC	1	Additional bilateral tubers seen
**Total = 47**		**Total = 13 with additional findings + 2 with 3T findings not re-demonstrated**	

*Ipsilateral/contralateral findings are relative to the 3T finding or the epilepsy lateralization; pts, patients; N/A, none applicable; FCD, focal cortical dysplasia; HS, hippocampal sclerosis; DVA, developmental venous anomaly; PVNH, periventricular nodular heterotopia; Cav Mal, cavernous malformation; PMG, polymicrogyria; TSC, tuberous sclerosis complex*.

### ICEEG and Surgery in the Overall Cohort

Of the 47 patients in the overall cohort, 34 patients underwent surgery, and 18 of them had focal lesions such as HS, FCD, and tumor. In these 18 patients, surgery was typically performed without ICEEG (only 22%, 4 of 18 had ICEEG) and included a complete resection of the lesion. For the four patients who did undergo ICEEG, the relationship between lesion and ICEEG ictal onset was concordant in three and concordant-minus in one. Seizure freedom was achieved in 78% of these patients (14/18, mean follow up = 2.2 years).

Out of the 34 patients who underwent surgery in the overall cohort, 16 patients had more widespread/multifocal lesions or lesions with unclear epileptogenicity, such as PVNH, PMG, infarct, TSC, cysts and vascular malformations, surgery was typically performed following ICEEG (75%, 12 of 16 had ICEEG); the relationship between lesion extent and ICEEG ictal onset was quite variable: concordant-plus in 4, partial in 5 and discordant in 3. The surgical strategy was largely based on ICEEG results rather than on the extent of the MRI-defined abnormalities. Seizure freedom was seen in 56% of patients (9/16, mean follow up = 1.8 years). Notably, the additional smaller abnormalities identified by 7T did not all carry epileptogenicity, and in only one patient (shown in Figure 1) was an additional small heterotopia near the amygdala confirmed to be part of the ictal onset zone on ICEEG.

### Radiologist Review for 40 Patients (47 Lesions)

Out of the overall cohort, a subset of 40 patients (47 lesions, as five patients had multiple lesions that were reviewed separately) underwent paired 7T−3T review by the seven neuroradiologists.

**Figure 3:**
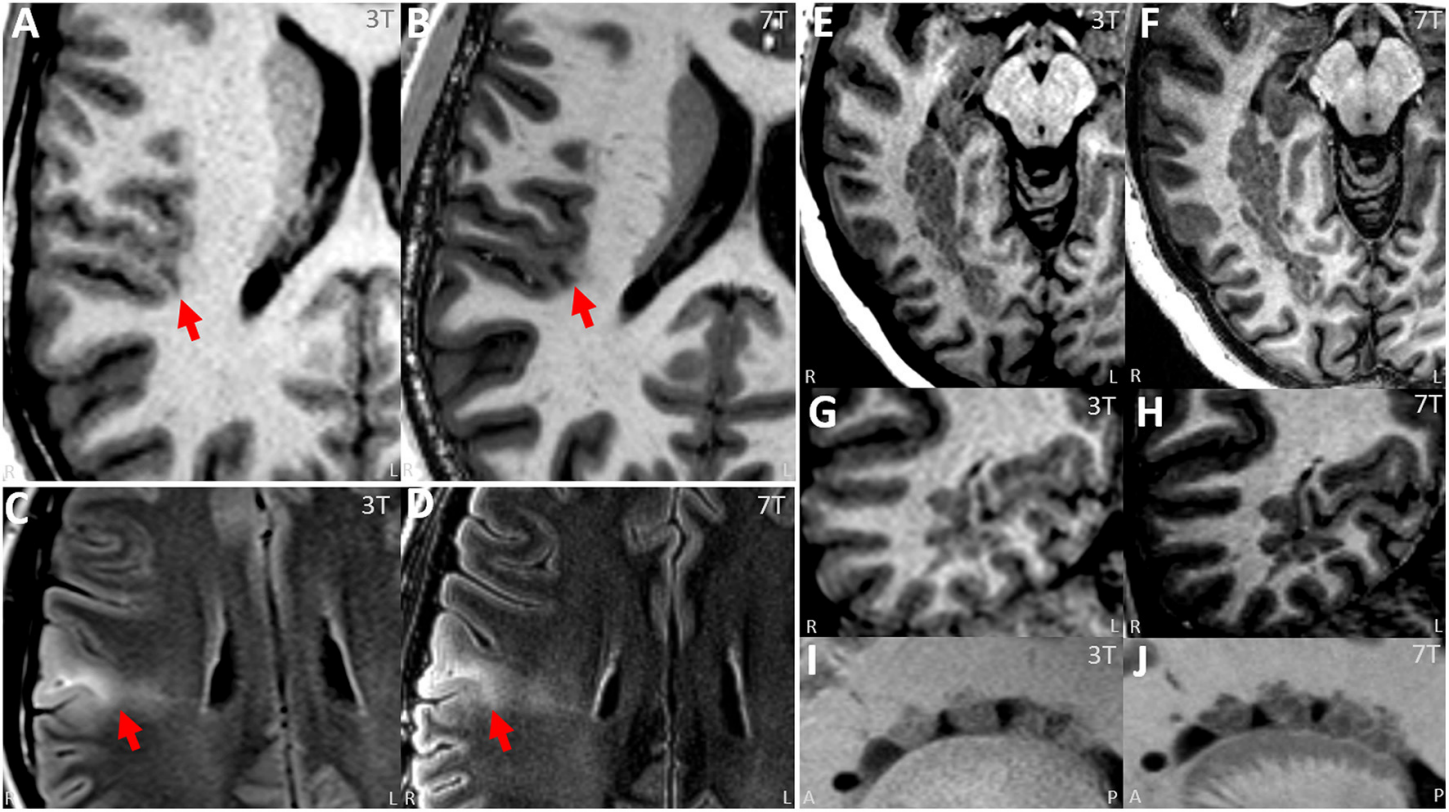
**(A,B)** Paired 3T−7T Images from a patient with histologically confirmed FCD IIB showing similar lesion presence, improved lesion conspicuity and improved boundary from the neuroradiologist team assessment. **(A)** Images from T1w MPRAGE sequence from 3T with arrow pinpointing the lesion. **(B)** Images from T1w MP2RAGE sequence from 7T at the same location, with arrow pinpointing the lesion. **(C,D)** Paired 3T−7T Images from another patient with histologically confirmed FCD IIB showing similar lesion presence, improved lesion conspicuity and improved boundary from the neuroradiologist team. **(C)** Images from T2w axial FLAIR sequence from 3T with arrow pinpointing the lesion. **(D)** Images from T2w axial FLAIR sequence from 7T at the same location, with arrow pinpointing the lesion. **(E–J)** Paired 3T−7T Images from a patient with nodular heterotopia showing similar lesion presence, improved lesion conspicuity and improved boundary from the neuroradiologist team. **(E,G,I)** Images from T1w MPRAGE sequence from 3T showing the lesion. **(F,H,J)** Images from T1w MP2RAGE sequences from 7T at the same location. **(E,F)** Axial zoomed, **(G,H)** coronal zoomed, and **(I,J)** sagittal zoomed.

**Figure 4:**
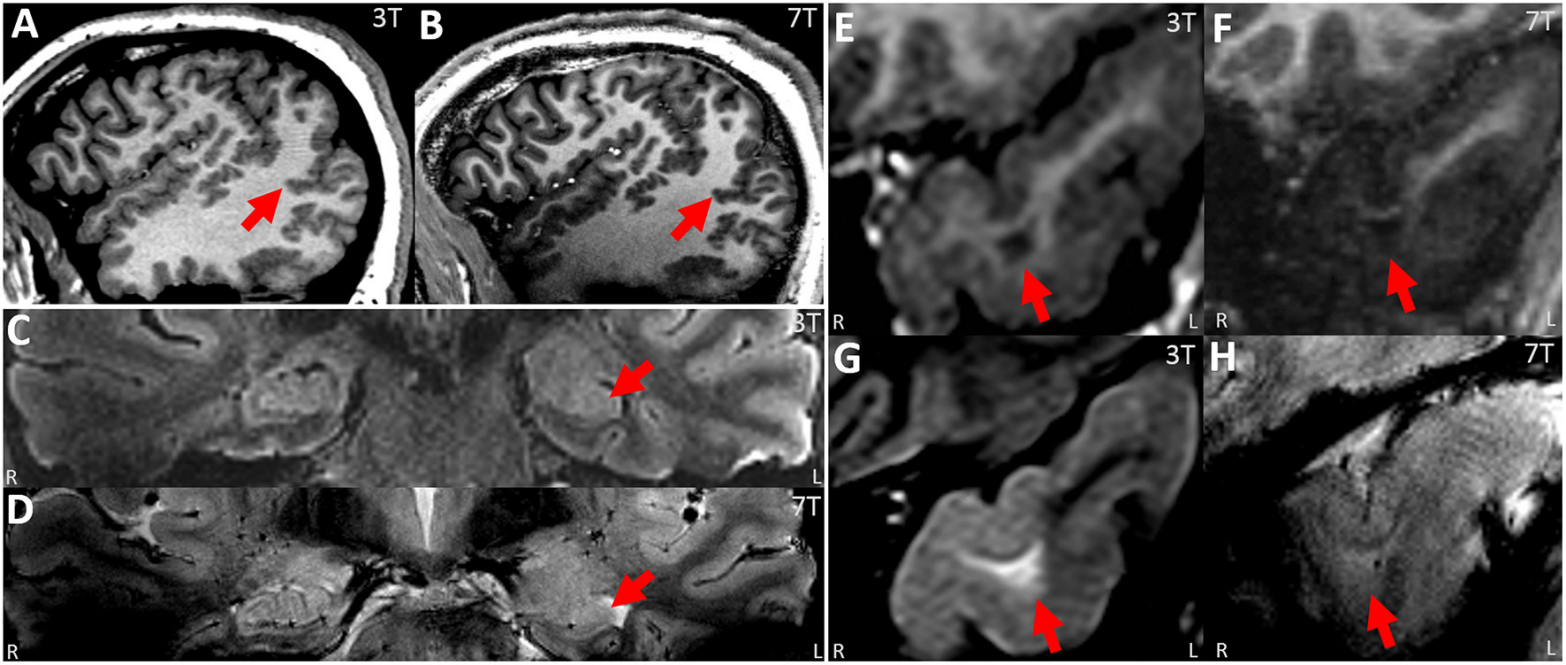
**(A,B)** Paired 3T−7T Images from a patient with extensive PMG in the right temporo-parieto-occipital region, showing similar lesion presence, improved lesion conspicuity and improved boundary (especially at the posterior part of the lesion, as shown by arrow) as assessed by the neuroradiologist team. **(A)** Sagittal image from T1w MPRAGE at 3T. **(B)** Sagittal image from T1w MP2RAGE at 7T. **(C,D)** paired 3T−7T Images from a patient with histologically confirmed HS type I in the left hippocampus (pointed out by arrow). For this patient, lesion presence, conspicuity and boundary were scored to be similar by the neuroradiologist team. Although the internal structures within the hippocampus might be better visualized at 7T, this improvement was mitigated by the loss of signal in the immediately adjacent temporal lobe (therefore more artifacts were scored to be present by the team). **(C)** T2w coronal FLAIR at 3T; **(D)** T2*w coronal GRE from 7T at the same position. **(E–H)** Paired 3T−7T Images from a patient with histologically confirmed FCD type IIb in the left temporal pole. Lesion presence, conspicuity and boundary were scored to be better at 3T from the neuroradiologist team. **(E,G)** Coronal zoomed images from T1w MPRAGE/T2w FLAIR sequences from 3T showing the lesion. **(F,H)** coronal zoomed images from T1w MP2RAGE/T2*w GRE sequences from 7T at the same location.

**Figure 5:**
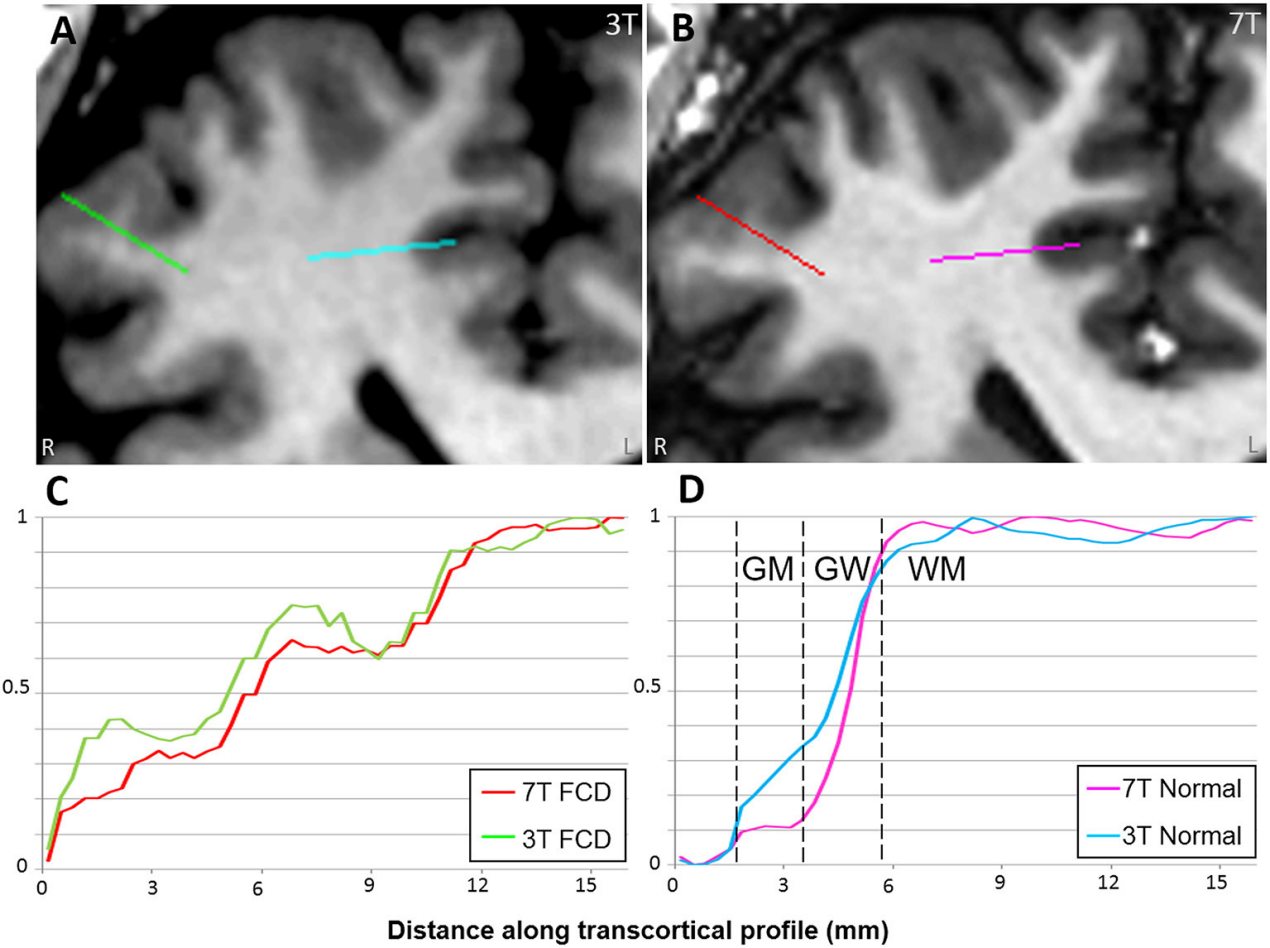
Comparison of MRI signal intensity plots along transcortical profiles obtained from FCD and normal cortex in a patient with histopathologically confirmed FCD type IIb. 3T T1w MPRAGE **(A)** and 7T T1w MP2RAGE **(B)** were used for the comparison. Signals are plotted across the transcortical profiles shown on the MRI (starting from GM and ending at WM). Zero was set as the mean signal from CSF. Signals were normalized with their min and max values. In the normal cortex **(D)**, GM, WM, and gray-white transition (GW) can be clearly distinguished from the 7T signal plot. In the FCD cortex **(C)**, such distinction is absent due to GW blurring.

1. *Presence and diagnosis of lesion* (0 = absent, 1 = equivocal, and 2 = present)
  As shown in Table 2, in the overall group, on a scale from 0 to 2, 3T had an average score of 1.43 (95% CI: 1.29, 1.58); 7T had an average score of 1.37 (95% CI: 1.23, 1.51). There was no significant difference regarding recognizing the presented lesion demarked by the provided key images when comparing 3T and 7T. Similarly, in each pathology subgroup, no significant differences were seen. Chance-adjusted agreement was within the fair agreement range for the review of both 3T (Kappa = 0.29, 95% CI: 0.20,0.39) and 7T scans (Kappa = 0.25, 95% CI: 0.15,0.34), which was not significantly different. In terms of lesion diagnosis, when the lesion was rated to be present (score ≥ 1) at 3T, 95% of diagnoses were consistent with the initial radiologist review; at 7T the percentage of consistent diagnoses slightly increased to 96% which was not significantly different. Therefore, overall, the visual presence or diagnosis of purposely demarked lesions was not different between 3T and 7T.
2. *Conspicuity of lesion* (−2 = 3T definitely better, −1 = 3T mildly better, 0 = equal; 1 = 7T mildly better; 2 = 7T definitely better)
  As shown in Table 3, in the overall cohort, neuroradiologists found 7T to be superior 43.8% of the time, and 3T to be superior 14% of the time (*p* < 0.001). The subgroup differences were significant for PVNH (*p* < 0.001), FCD (*p* = 0.035), and PMG (*p* = 0.012), but not for HS or tumor. Chance-adjusted agreement was within the fair agreement range (Kappa = 0.31, 95% CI: 0.18,0.47). Examples of the assessment for lesion conspicuity are illustrated in Figure 3 (FCD and PNVH), and Figures 4A,B (PMG), where improvement of conspicuity was seen at 7T. An example of HS where lesion conspicuity was rated to be similar between 3T and 7T is shown in Figures 4C,D. Figures 4E–H also contains a counterexample where 3T was rated to show better conspicuity than 7T (FCD in temporal pole) due to susceptibility artifacts affecting the 7T scan, particularly the T2*-weighted images (Figure 4H).
3. *Boundary of lesion* (0 = not clearly visible; 1 = clearly visible)
  As shown in Table 3, in the overall group, 69% of the cases at 7T were scored as “lesion boundary clearly visible,” compared with 58.4% of cases at 3T (*p* = 0.003). The subgroup differences were significant for PVNH (*p* < 0.001), tumors (*p* < 0.001), and PMG (*p* < 0.001), but not for FCD or HS. Chance-adjusted agreement was within the fair agreement range for the review of both 3T (Kappa = 0.35, 95% CI: 0.21,0.49) and 7T scans (Kappa = 0.39, 95% CI: 0.24,0.53), which was not significantly different. Examples of lesion boundary assessment can be seen in Figures 3, 4.
4. *Gray-white tissue contrast* (−2 = 3T definitely better, −1 = 3T mildly better, 0 = equal, 1 = 7T mildly better, and 2 = 7T definitely better)
  Comparison of normal gray-white boundary conspicuity on T1-weighted images was significantly better at 7T (*p* < 0.001). 7T was superior on 90.0% of scans (CI: 86.0, 93.0%), and 3T was superior in only 4.6% of scans (CI: 2.8, 7.5%). An example of this assessment is illustrated in Figure 5, which shows the MRI signal intensity plots obtained with 7T and 3T from the same patient's normal and dysplastic cortex. In the normal cortex (Figure 5D), GM, WM and gray-white transition (GW) can be clearly distinguished from the 7T signal plot. In the FCD cortex Figure 5C), such distinction is absent due to GW blurring. The steepness of the slope of signal increase observed in normal cortex is rapid (Figure 5D); while the dysplastic cortex shows a more gradual signal change (Figure 5C). As best seen for the normal cortex (Figure 5D), the mean GM signal is much less than that of WM at 7T, indicating increased contrast; in addition, the slope of signal increase is much greater, indicating sharper gray-white boundary.
5. Artifacts
  In the overall cohort, artifacts affected lesion conspicuity in 28.3% (CI: 20.5%, 37.6%) of scans at 7T, compared to 4.9% of scans at 3T (CI: 2.6%, 9.0%, *p* < 0.001). The artifact location was most prevalent in the antero–inferior temporal lobes, where artifact types such as B_1_ field inhomogeneity (Figures 4E–H), motion and pulsation were cited by at least one reader in more than 50% of scans.
6. Most helpful sequence for diagnosis
  On 3T, FLAIR was rated as the most helpful sequence overall (41.3%), and was also the most helpful sequence in tumor (85.7%), HS (61.2%), and FCD (51.8%); MPRAGE was rated as the most helpful sequence in PVNH (95.2%) and PMG (80.0%).
  On 7T, MP2RAGE was rated as the most helpful sequence overall (49.8%), likely due to its exquisite gray-white distinction both in terms of spatial resolution and contrast-to-noise ratio. MP2RAGE was also the most helpful sequence in FCD (49.1%), PMG (88.6%), and PVNH (97.6%); FLAIR was rated to be the most helpful sequence in HS (53.1%) and tumor (71.4%).

**Table 2:** Comparison statistics for presence of lesion (0 = absent, 1 = equivocal, and 2 = present).

	**Mean (95% CI)**			
**Group**	**3T**	**7T**	**Difference**	***P*-value^a^**
All	1.43 (1.29,1.58)	1.37 (1.23,1.51)	0.06 (−0.01,0.14)	0.059
FCD	1.83 (1.51,2.15)	1.68 (1.36,2.00)	0.15 (−0.01,0.31)	0.10
HS	1.47 (1.11,1.83)	1.43 (1.07,1.79)	0.04 (−0.19,0.27)	0.26
PVNH	1.14 (0.90,1.39)	1.12 (0.88,1.36)	0.02 (−0.13,0.18)	0.32
Tumor	1.80 (1.60,2.08)	1.80 (1.68,2.01)	0.00 (−0.08,0.08)	0.99
PMG	1.31(1.18,1.45)	1.14 (1.01, 1.28)	0.17(−0.08,0.42)	0.14

a *P-value from proportional odds logistic model comparing 3T and 7T*.

**Table 3:** Comparison statistics for lesion conspicuity and lesion boundary.

	**Lesion conspicuity**			**Lesion boundary**		
**Groups**	**7T Superior (%)**	**3T Superior (%)**	***P*-value^a^**	**7T (%)**	**3T (%)**	***P*-value^b^**
All	43.8 (36.0,51.9)	14.0 (8.6,22.0)	<0.001	69.0 (59.3,77.3)	58.4 (48.8,67.3)	0.003
FCD	43.8 (31.7, 56.6)	17.0 (8.5,31.0)	0.035	45.5 (29.6,62.4)	33.9 (21.3,49.4)	0.059
HS	36.7 (23.5, 52.3)	26.5 (13.5,45.6)	0.53	61.2 (44.9,75.4)	57.1 (31.6,79.4)	0.69
PVNH	71.4 (61.3, 79.8)	0 (NA)	<0.001	88.1 (73.6,95.2)	69.0 (53.8,81.0)	<0.001
Tumor	17.1 (8.0,32.9)	20.0 (5.5,51.6)	0.92	80.0 (55.4,92.8)	65.7 (39.2,85.1)	<0.001
PMG	17.1 (8.0, 32.9)	0 (NA)	0.012	88.6 (82.5,92.7)	60.0 (50.4,68.9)	<0.001

a *P-value from linear mixed model comparing level against equality of 3T and 7T*.

b *P-value from logistic regression model with generalized estimating equations comparing 3T and 7T*.

*For lesion conspicuity, the percentage is calculated on the sum of “definitely better” lesions and “mildly better” lesions over the total number of lesions in each category. For lesion boundary, the percentage is calculated by the number of lesions scored as “lesion boundary clearly visible” divided by the number of all lesions in each category*.

## Discussion

We present a large cohort of 47 pharmacoresistant focal epilepsy patients evaluated with 7T MRI who already had lesion(s) reported on the 3T MRI. Prospective review was performed to identify the clinical yield of 7T in terms of the additional findings it could provide, and retrospective review was performed by a team of neuroradiologists to examine agreement statistics of lesion characteristics. Key findings from our study include: (1) in 68% of the cases, no new lesion was detected on the 7T as compared to the 3T, indicating that 7T is likely not going to replace 3T for *all* the patients undergoing presurgical evaluation, (2) the main detection yield for additional findings at 7T was seen in the patients who had already extensive or multifocal abnormalities such as PMG, PVNH, perinatal infarct and TSC; for these patients, additional smaller abnormalities were frequently seen on the ipsilateral or the contralateral side, (3) occasionally, 7T could lead to detection of additional FCD or could be used to strengthen/rule out ambiguous findings on 3T, (4) agreement data from the neuroradiologist team review show that 7T was mildly superior to 3T for lesion conspicuity and lesion margins, particularly for FCD, PVNH, and PMG; 7T also showed significantly improved gray-white distinction, but had significantly more artifacts, particularly in the antero-basal temporal lobe.

### Lesion Detection Yield

In patients with large or multifocal malformations such as PMG, PVNH, infarct, and TSC, the extent of the lesions might be underestimated especially on the contralateral side (16, 17). Our data showed that 7T provides improved anatomical delineation of the extent and/or the bilateral nature of some large lesions, as illustrated in 19% of the patients in our cohort. Our findings are consistent with previously published data on the uncovering of bilateral abnormalities on 7T in 4/6 patients who were shown to harbor only unilateral PMG on 3T (16). Of note, our data suggest that in the setting of an extensive cortical malformation such as PMG or PVNH, all the lesions do not necessarily have the same epileptogenicity and guidance of resection should come from ICEEG, not merely MRI. This is consistent with prior studies (18, 19).

In patients with more widespread/multifocal lesions or lesions with unclear epileptogenicity, such as PVNH, PMG, chronic infarct and TSC, surgery was typically performed following ICEEG and the ICEEG ictal onset may be broad, diffuse, or simultaneously involving multiple brain regions, reflecting the complexity of managing these cases. This was seen in the 5 patients for whom the relationship between 7T lesion(s) and ICEEG was classified as partial or concordant-plus (lesion extent > ICEEG ictal onset). Discrepant ICEEG onset and lesion locations were seen in three patients, including one patient with hippocampal cysts (expected), one with DVA (expected), and one with PMG [not unexpected due to the heterogenous epileptogenicity of PMG which may extend to its adjacent/distant cortex, as reported by previous studies (19, 20)].

Overall, the additional findings noted on 7T, such as the additional smaller abnormalities in the case of extensive or multifocal abnormalities, can be used as potential targets to improve the accuracy of ICEEG implantation, if they concur with the patient's electroclinical profile, as illustrated in the patient with PVNH in Figure 1. The additional detection of FCD could be particularly relevant (although it occurred only once in this cohort). As reported in our recent study focusing on the efficacy of 7T in 3T-negative patients, the 7T-detected subtle FCD lesions were highly relevant to the epilepsy and needed to be included in the surgical resection for seizure freedom (10).

In two cases in our cohort, the lesions on 3T were not recapitulated on 7T due to artifacts and partial volume effects at 3T, respectively. Subsequent ICEEG/surgery performed was consistent with the 7T impression, supporting the validity of the additional information generated by 7T. It is worth pointing out that refuting false positive findings does carry clinical significance, as they may misguide the next steps of clinical decision making, such as surgical candidacy, need for ICEEG and resection extent.

### Lesion Presence, Diagnosis, Conspicuity, and Boundary

The advantage of 7T compared to 3T stems from its higher spatial resolution (for almost all the comparable sequences in the two protocols), signal-to-noise ratio and contrast-to-noise ratio (as illustrated in Figure 5). Partial volume effects are likely reduced due to the improved in-plane resolution and slice thickness. This was consistent with our results on the systematic scoring performed by multiple neuroradiologists with the same full DICOM viewer used in clinical practice. We first showed that 7T review was consistent with 3T review in terms of the presence and diagnosis of the lesion, when the lesion was pointed out on provided key images. This finding provides a sanity check for the other lesion characteristics' scoring. We then showed that 7T was superior to 3T for lesion conspicuity, lesion margins and normal GW distinction. There were differences when different pathology groups were examined: FCD, PVNH and PMG benefited more often from 7T than did HS.

The advantage of 7T for FCD, for example, was likely due to the improved gray-white delineation for the cortical convexity, which sets the lesional cortex farther away from normal baseline, as shown in Figure 5. Our finding is supported by previous studies in the literature. Colon et al. reported a three-reviewer comparison between 7T and 3T for eight patients with suspected FCD, showing that 7T MRI scored significantly better for lesion conspicuity and demarcation; common radiological characteristics of FCD were separately rated and significance was seen in features such as GW blurring, abnormal internal structure and transition to normal cortex (7).

De Ciantis et al. (16) reported 10 patients with known PMG on 3T and demonstrated that 7T showed more extensive areas of PMG in 4 patients, due to the use of T2* susceptibility-weighted images that showed increased dilated superficial veins. Consistent with this study, our results showed robust improved visualization of PMG (by utilizing all the available sequences including T2*- and T1-weighted) in terms of conspicuity and boundary.

In terms of HS, although prior studies have shown efficacy of *in vivo* 7T to segment the subfields of hippocampi using manual or automated methods (21), in the current study we did not ask the neuroradiologists to comment on the hippocampal subfields to differentiate subtypes of HS, due to the fact that none of the patients had HS type II.

### Agreement

The superior spatial resolution of 7T led to smaller findings that may be normal variants and irrelevant to epilepsy, as reported by a recent study detailing normal variants on 7T images acquired from normal controls (8). This is exacerbated by artifacts that were more frequently encountered, as shown by our data. These aspects explain our finding that chance-adjusted Kappa agreements were all in the fair range for lesion presence, conspicuity, and boundary. This was also observed in a previous study (3). Few previous studies reported the actual agreement data on 3T−7T comparison studies. In a study comparing 40 sets of 7T and 3T brain images (including 9 epilepsy cases) (2), interrater agreement was as high as 93.3% at 7T, as compared to 69.7% at 3T. This difference was likely due to a much lower number of neuroradiologists involved in reviewing images (*N* = 2), as compared to the current study (*N* = 7). In fact, likely due to the large number of reviewers and fine-grained categories of the scoring system, the kappa agreement data on 3T was also in the fair range in our study, which is similar to the 7T agreement data. Therefore, the fair range perhaps reflects the human reviewer variabilities more than the imaging technique itself.

### Artifacts

Our data demonstrate that the current 7T MRI hardware has significantly more artifacts compared to 3T, which may have clinical implications. Because smaller voxel size amplifies sensitivity to motion, added cooperation from patients is required and should be emphasized during the entire scanning. With the current setup, the artifacts are more commonly affecting the temporal lobe regions, suggesting that patients with temporal lobe epilepsy, especially with suspected anterior–inferior temporal pathology, are likely to have a lower yield at 7T due to increased susceptibility and B_1_ inhomogeneity artifacts. These artifacts can hamper the recognition of structural changes associated with temporal lobe epilepsy, such as the collateral sulcus and blurring of the temporal pole (22). Future advances with gradient coils with higher order shims, as well as more advanced pulse sequence design combined with parallel transmit parallel transmit (PTx) systems, can markedly improve image quality (23, 24), in particular involving the anterior–inferior temporal lobes. Encouraging results were already shown by recent studies that adapted 7T sequences on PTx to recover regions of low signal without increasing imaging time (25).

### Methodological Considerations

Recognizing the intrinsic limitations of comparing cross-field-strength studies, caution is needed to interpret these results as they could be dependent on the sequences included in the protocols and the choice of parameters for the acquisitions. 3T MRI is relatively highly optimized due to its wide usage in epilepsy evaluation, whereas the 7T MRI protocol still awaits further optimization. Our study, therefore, represents a snapshot comparison based on a given set of protocols, rather than a definitive cross-sectional comparison. Nevertheless, our data showed the efficacy of 7T in a subset of patients (FCD, PVNH and PMG) and highlighted careful consideration of the epileptogenicity of the additional findings, despite the relatively early stage of 7T optimization as compared to 3T. Future improvements yielding higher-quality 7T images over the whole brain should further improve its performance.

### Limitations

1. Our cohort did not include every 3T-lesional patient evaluated for epilepsy surgery during the course of the study. Generalization of our results to all patients undergoing pre-surgical evaluation therefore requires caution.
2. There was only one neuroradiologist who performed the prospective review, although the 7T findings were re-reviewed and discussed at the PMC, which included consensus opinion from another neuroradiologist.
3. Although the majority of the sequences for the MRI protocols at 3T and 7T overlapped, there were some differences: T2 TSE images were initially unavailable for 7T, and T2* GRE was not clinically obtained at 3T in our standard-of-care epilepsy protocol. Among the technical reasons leading to these differences are more stringent SAR limitations at 7T, particularly affecting the whole-brain TSE sequence, which only became available later into the study. While it is ideal that a comparative study with exactly the same sequences should have been used, our 7T and 3T protocols did overlap as much as possible. Data from this study, as preliminary findings, will allow future studies using more optimized/consistent protocols for comparison. Overall, caution should be used before generalizing results from our study to all 7T applications.

## Conclusions

For patients with pharmacoresistant epilepsies and known lesion(s) on the 3T MRI, 7T MRI may better elucidate the extent of multifocal abnormalities such as PVNH and PMG. The additional findings seen on 7T can be used as potential targets to improve the accuracy of ICEEG implantation, in addition to planning margins for respective surgery. The subset of patients with FCD, PVNH and PMG would likely benefit the most from 7T for an increased lesion conspicuity and lesion boundary, while pathologies in the antero-inferior temporal regions benefit less due to artifacts. Future improvements yielding higher-quality images over the whole brain are necessary to further elevate the performance of 7T for patients with pharmacoresistant epilepsies.

## Data Availability Statement

The datasets presented in this article are not readily available because they may contain information that could compromise the privacy of research participants. Requests to access the anonymized datasets should be directed to the corresponding author.

## Ethics Statement

The studies involving human participants were reviewed and approved by Cleveland Clinic institutional review board. Written informed consent to participate in this study was provided by the participants or their legally authorized representatives.

## Author Contributions

ZIW: study concept and design, MRI data collection, MRI data analysis, clinical and imaging data interpretation, manuscript drafting, critical revision, and study supervision. S-HO and MLo: MRI data collection. MLa, PR, VH, VS, DM, JL, and TE: MRI review. JB: statistical analysis. IB: pathology examination. JG-M and WB: intracranial EEG implantation and interpretation, laser ablation and resective procedures. IN: clinical and imaging data interpretation, critical revision. SEJ: study concept and design, MRI review, clinical and imaging data interpretation, critical revision, and study supervision. All authors contributed to the article and approved the submitted version.

## Conflict of Interest

IN is on the Speakers' bureau of Eisai. JG-M received educational grant from Zimmer Biomed. SJ received travel and speaker fees from SIEMENS Healthineers. The remaining authors declare that the research was conducted in the absence of any commercial or financial relationships that could be construed as a potential conflict of interest.

## Supplementary Material

Supporting Material 1 Detailed patient demographics.
