# Supplementary material for: Radiological and Clinical Value of 7T MRI for Evaluating 3T-Visible Lesions in Pharmacoresistant Focal Epilepsies
Source: Front Neurol. 2021 Mar 2;12:591586. doi: 10.3389/fneur.2021.591586 (PMC7960771; doi:10.3389/fneur.2021.591586)
Supplement: Supporting Material 1 — Detailed patient demographics. [file Data_Sheet_1.docx]

| **Pt #** | **Sex** | **Hand** | **ED (y)** | **Age (y)** | **Epilepsy type** | **Seizure Types (Semiology)** | **Seizure Frequency** | **EEG**  **interictal** | **EEG**  **ictal** | **PET** | **SPECT** | **MEG** | **ICEEG type (SEEG or subdural, # of electrodes, # of contacts)** | **ICEEG ictal onset** | **Surgery** | **Pathology** | **Seizure Outcome** | **3T** | **7T** | **7T lesion vs. ICEEG ictal onset** | **7T lesion included in resection?** |
| --- | --- | --- | --- | --- | --- | --- | --- | --- | --- | --- | --- | --- | --- | --- | --- | --- | --- | --- | --- | --- | --- |
| P1 | M | R | 24 | 25 | R T | Automotor -> L versive (head) -> GTC | 1 per day | IS: R T  SW: R T | R T | Subtle hypometabolism  R mes T  R insula  R FPop | Not done | Not done | Not done | Not done | R ATL including mesial structures | HS type II | SF  2.3 years | R HS | R HS | NA | Y |
| P2 | M | R | 6 | 51 | L T | Automotor  ->GTC | 1-5 per month | IS: L FT  SW: L FT | L FT | Subtle hypometabolism  L mes T | Not done | Negative | Not done | Not done | L ATL including mesial structures | FCD IIIa (inconclusive HS subtype due to limited tissue) | SF  3.4 years | L HS | L HS | NA | Y |
| P3 | F | R | 4 | 57 | R T | Aura (autonomic) -> Autonomic seizure | 1 per day | None | R T | Not done | L post sup T  L mes F  22 s | Not done | Not done | Not done | R ATL including mesial structures | HS type I | SF  2.1 years | R HS | R HS | NA | Y |
| P4 | F | R | 19 | 20 | L T | 1. Automotor  2. Aura (autonomic) -> Dialeptic | 3-10 per month | IS: L T  SW: L T | L T | Not done | Not done | Not done | Not done | NA | Not done | No surgery | NA | L HS | L HS | NA | NA |
| P5 | M | R | 24 | 33 | R FT | 1. Aura (visual/somatosensory/autonomic)  2. Autonomic -> Automotor  3. Autonomic seizure -> Automotor -> L versive -> GTC | 4-6 per month | CS: R T  SW: R T | R T | Subtle hypometabolism  R ant T | R post lat T  Bi post insular  Bi ant T  Bi dorsolateral FC  11 s | Negative | SEEG, 14 electrodes | R HIPPO  R post insula | R ATL + R post insular resection | HS Type I  Insular specimen negative | SF  1 year | R HS | R HS  R post insular FCD | C | Y |
| P6 | F | R | 20 | 22 | L T | Aura -> Automotor -> R version -> GTC | 3 per month | IS: L T  SW: L T | L FT | Moderate hypometabolism  L T | L insula  L ant T  L post lat T  19 s | Negative | SEEG, 13 electrodes | L mes T | L ATL including mesial structures | Negative | NSF  1 year  (recurrence at 11 months) | L HS | L HS | NA | Y |
| P7 | F | R | 15 | 31 | L TPO | 1. Aura(visual)  2. Dialeptic -> R Face Tonic -> R Face clonic -> GTC  3. Dialeptic -> R Eye versive -> Face clonic  4. Dialeptic | 3-20 per day | SPK: Lateralized LH max TPO  PSPK: Lateralized LH max TPO | Lateralized LH max TP | Moderate  Hypometabolism  L Fop/Top/Pop  L insula  Subtle hypometabolism  L mes T  L OF  L inf lat F | L post Lat T  L TP  L TO  20 s | Loose cluster:  L TO | SEEG, 15 electrodes | Diffuse, L TPO | Not done | No surgery | NA | L HS | Negative | NA | NA |
| P8 | F | L | 8 | 18 | R T | 1. Aura (somatosensory/  Abdominal/olfactory)  2.Aura (Abdominal/olfactory)  -> Autonomic -> Dialeptic -> L Face clonic  3. NCS | 5-6 per week | IS: R T  SW: R FT | R T | Subtle hypometabolism  Bi T (R>L) | Not done | Negative | Not done | Negative | R ATL including mesial structures | HS type I | SF  1.25 years | Bi HIPPO hyperintensity (R>L) | R HS | NA | Y |
| P9 | F | R | 10 | 38 | R T | Autonomic seizure | 1-7 per day | SW: R FT | Lateralized RH max FT | Subtle hypometabolism  Bi T (R>L) | Not done | Not done | Not done | NA | R ATL including mesial structures | Negative | SF  2 years | R HIPPO signal and volume increase | R HIPPO signal and volume increase | NA | Y |
| P10 | M | R | 21 | 26 | R FPop | 1. Automotor  2. Axial tonic -> Bilateral asymmetric Tonic -> R face and arm clonic | 1-2 per week | IS: L FT, R FT  SW: L F, L FT, R FT | L FT or nonlocalizable | Not done | Not done | Not done | Not done | Not done | R FPop lesionectomy | FCD IIb | SF  2.8 years | R FPop FCD | R FPop FCD | NA | Y |
| P11 | M | R | 13 | 29 | R F | 1. Dialeptic  2. Dialeptic -> Complex Motor  3.Dialeptic -> GTC  4. NCS | 1-3 per day | PSPK: L FC | L FC | Not done | Not done | Not done | SDG + depth  (4 plates,2 depths) | L pars orbitalis | L pars orbitalis lesionectomy | FCD IIb | SF  3.2 years | L pars orbitalis FCD | L pars orbitalis FCD | C | Y |
| P12 | M | R | 17 | 21 | R F | 1. Aura (L arm somatosensory)  -> L arm tonic -> GTC | 1-5 per day | No interictal | Nonlocalizable | Subtle hypometabolism  Bi T (L>R)  L parasagittal F  L ant F | Not done | Negative | Not done | Not done | R mid F lesionectomy | FCD IIa | SF  3.3 years | R mid F FCD | R mid F FCD | NA | Y |
| P13 | M | R | 10 | 19 | R P | 1. Aura (Psychic)  2. Aura (Psychic) -> L versive -> GTC  3. Aura (visual) -> bilateral asymmetric tonic -> GTC | Daily | SWC: Gen  SW: R TP, R F, Bi F | 1. Lateralized RH, max R P and vertex  2. R P | Subtle hypometabolism  Bi T (L>R)  L post P  L post PO | Nonlocalizable | Loose cluster: R SPL | SEEG, 16 electrodes | L mes P | R mes P resection | FCD IIa | NSF  2.5 years  (recurrence at 10 months, Class II) | L lat P  FCD | Negative | NA | NA |
| P14 | M | R | 11 | 21 | L T | Dialeptic -> Automotor | 1-2 per month | SW: R T, L T | L T | Subtle hypometabolism  Bi T (L>R)  Bi F  Bi Pop (L>R) | Not done | Not done | Not done | Not done | L ATL sparing mesial structures | FCD IIb | SF  2.5 years | L ant T  FCD | L ant T  FCD | NA | Y |
| P15 | F | R | 15 | 16 | L F | 1. NCS -> Bilateral Asymmetric Tonic -> Bilateral Asymmetric Clonic  2. Myoclonic | 2-3 per week | CS: L CP and vertex  SPK: Vertex and L C  SWC: Gen | 1. Vertex and L C  2. Gen | Subtle hypometabolism  Bi T  Moderate to severe  Hypometabolism  R basal post T | Not done | Scatter:  L C, L F  Loose cluster:  R C | Not done | Not done | L SFG lesionectomy | FCD IIa | SF  2.5 years | L SFG FCD | L SFG FCD | NA | Y |
| P16 | F | Ambidextrous | 1 | 14 | L C | 1. Epilepsia partialis continua -> R hand and arm myoclonic  2. R mouth and arm tonic -> R arm clonic | 4-5 per week | CS: L C  SPK: L Parasagittal and vertex | L Parasagittal and vertex | Not done | Not done | Not done | Not done | NA | Not done | No surgery | NA | L C  FCD | L C  FCD | NA | NA |
| P17 | F | R | 6 | 23 | LH | 1. Psychic aura -> R face tonic -> GTC  2. Aphasic -> GTC | 1-2 per month | CS: L TP  IS: Gen and max L TPO  SW: MF LH  PSPK: MF LH | L TP | Subtle hypometabolism  R inf O  R OF  L lat TP | L ant insula  L inf basal F  13 s | Loose clusters:  L mid post T  L inf P  L inf F | SEEG, 14 electrodes | L basolateral T  L IPL | Not done | No surgery | NA | L basolateral T FCD | L basolateral T FCD | C- | NA |
| P18 | M | R | 19 | 24 | R O | 1. Aura (Visual) -> Tonic -> GTC  2. Axial tonic -> L eye version -> GTC | 1-2 per month | CS: R PO  IRS: R PO  SPK: R PO | R PO | Subtle hypometabolism  R lat O | Attempted but only baseline | Tight cluster:  R basolateral O | SDG (5 plates) | R lat O | R O resection | FCD IIb | SF  1 year | R lat O FCD | R lat O FCD | C | Y |
| P19 | M | R | 32 | 32 | R TP | 1. Aura (somatosensory R hand, L leg or Gen)  2. Aura (somatosensory) -> Dialeptic -> L face versive -> GTC  3. L face versive -> GTC | 2-3 per week | SW: R FT | R TP | Not done | Not done | Not done | Not done | Not done | R P lesionectomy and R ATL including mesial structures | R P: FCD IIB  R HIPPO: HS type I | NSF  2 years (recurrence at 5 months, Class II) | R P FCD  R HS | R P FCD  R HS | NA | Y |
| P20 | F | R | 11 | 26 | L P | Dialeptic -> R Versive -> GTC | 4-5 per month | IS: Gen  SW: L FT (80%), L T (10%), L F (10%) | L CT | Not done | Not done | Tight cluster:  L TPO | Not done | Not done | L SMG lesionectomy | DNET, grade I | SF  2.8 years | L SMG low grade neoplasm | L SMG low grade neoplasm | NA | Y |
| P21 | M | R | 5 | 25 | L T | 1. Aura (gustatory) -> automotor  2. GTC | 1. 1-2 per week  2. Total 4 GTC per lifetime | IS: Lateralized LH max L T | None recorded | Not done | Not done | Not done | Not done | Not done | L parahippocampal lesion laser ablation | No tissue sent (laser) | SF  1.5 years | L parahippocampal low grade neoplasm | L parahippocampal low grade neoplasm | NA | Y |
| P22 | F | R | 17 | 33 | L T | 1. Aura (auditory/ psychic/gustatory)  2. Aura (auditory) -> Dialeptic -> GTC | 1 per week | CS: LT    IRS: max L FT  SW: LT | Lateralized LH max L FT | Not done | Not done | Not done | Not done | Not done | L sup/mid T lesionectomy | Ganglioglioma, grade I | NSF  2.8 years  (recurrence at 2 months; Class III) | L sup/mid T low grade neoplasm | L sup/mid T low grade neoplasm | NA | Y |
| P23 | F | R | 15 | 49 | R F | Aura (somatosensory lip and tongue) | 1-5 per month | IRS: Gen max bi F or FT  SW: Bi F | No EEG change | Not done | Not done | Not done | Not done | Not done | Not done | No surgery | NA | R mes F low grade neoplasm | R mes F low grade neoplasm | NA | NA |
| P24 | F | R | 26 | 32 | LH | R head version -> Gen tonic | 1 per year | Normal | None recorded | Not done | Not done | Not done | Not done | Not done | Not done | No surgery | NA | L mid F low grade neoplasm | L mid F low grade neoplasm | NA | NA |
| P25 | F | R | 9 | 23 | R T | Aura (psychic/ autonomic) -> L face versive -> GTC | 2-3 per month | CS: R T  SW: R T | R TP | Subtle hypometabolism  Bi T (R>L)  R lat T  R lat O  R mes TO  R L P  R FTop | R post STS  R post insula  R post HIPPO  R Mes TO  L Lat TP  30 s | Scatters:  R perisylvian  R post TPO | SEEG, 19 electrodes | R post STS | R lat T resection including STG | Negative | NSF  1.5 years  (recurrence within 1 week; Class IV) | Cysts L HIPPO | Cysts L and R HIPPO | D | N |
| P26 | F | R | 9 | 37 | L FT | NCS -> R face tonic -> R eye versive -> GTC | 1-2 per month | IS: Gen, Lateralized RH, or Lateralized LH  SW: L T | L FT | Subtle hypometabolism  R OF  R ant dorsal med parasagittal F  L lat P | Not done | Not done | Not done | Not done | Not done | No surgery | NA | L P Cav Mal | L P Cav Mal | NA | NA |
| P27 | F | R | 2 | 46 | L T | Aura (psychic) -> Aphasic -> Dialeptic | 1 per week | None | L TP | Not done | Not done | Not done | Not done | Not done | Not done | Cav Mal | SF  1.3 years | L T Cav Mal | L T Cav Mal | NA | Y |
| P28 | M | R | 14 | 64 | Bi FT | 1. Dialeptic-> Axial tonic -> Automotor  2. Dialeptic -> Bilateral Asymmetric tonic -> Axial tonic | 3-4 per month | IS: Gen, Bi F, Bi T  SW: R F, R FT, L FT | Nonlocalizable | Negative | Attempted but only baseline | Tight clusters:  L mes T  R mes T  L OF | Not done | Not done | Not done | No surgery | NA | L F DVA | L F DVA with R F microbleed | NA | NA |
| P29 | M | L | 24 | 25 | R T | 1. Aura (L leg somatosensory)  2. L face versive -> Bilateral asymmetric tonic -> GTC | 1-2 clusters per week | CS: R FT  IRS: Gen or R FT  SW: Bi F, R FT  SPK: R FT | R FT  R TP | Subtle hypometabolism  Bi T (R>L)  R mid and sup F | R ant med T  R mid lat F  7 s | Loose clusters:  L ant/lat T  R post perisylvian  R mes T | SEEG, 13 electrodes | R AMY | R ATL including mesial structures | Negative | NSF  2.5 years  (recurrence at 2 months; Class III) | R P DVA | R P DVA | D | N |
| P30 | F | R | 5 | 20 | RH | Dialeptic | 1-3 per month | IRS: L FT  SPK: Gen max L | Nonlocalizable (max Bi F) | Subtle  hypometabolism Bi T(L>R)  Bi P and PO (R>L) | Attempted but only baseline | Scatters:  R PO  L PO | Not done | Not done | R F lesionectomy including DVA and R OF resection | Venous angioma  mMCD II | NSF  1 year  (recurrence at 1 month; Class III) | R F DVA  R OF FCD | R F DVA  R OF FCD | NA | P |
| P31 | M | R | 8 | 45 | R FT | L eye tonic -> autonomic -> Bilateral asymmetric tonic -> GTC | 1 per month | IS: LT, RT | Nonlocalizable | Mild hypometabolism  Bi T  R ant T | R FTop  R post ins  7s | Nonlocalizable | SEEG, 16 electrodes | R HIPPO  R lat ventricle PVNH | R lat ventricle PVNH laser ablation | No tissue sent (laser) | NSF  1 year  (recurrence at 2 months; Class III) | R lat ventricle PVNH | R lat ventricle PVNH | C- | Y |
| P32 | F | R | 5 | 36 | RH | 1. Aura (nonspecific or somatosensory)  2. Dialeptic  3. NCS | 5-10 per day | IRS: Lateralized RH max CTP  SW: R FT, Bi O | Bi O  Lateralized RH | Subtle hypometabolism  Lat mid F | R basal med TO  R mid parasagittal F  R post dorsal F  L lat P  26 s | Tight cluster:  R Fop  Scatter:  R lat FP | SEEG, 16 electrodes | R lat ventricle PVNH  R IPL / R ITG / R IOG | R lat ventricle heterotopia laser ablation | No tissue sent (laser) | SF  3.5 years | Bi PVNH | Bi PVNH | P | P |
| P33 | F | R | 7 | 26 | R TO | 1. Aura (visual) -> complex motor -> GTC  2. Aura (visual) | 2-3 per week | IS: Gen, R T  SW: R TP  PSPK: R TP | Lateralized RH | Subtle hypometabolism  Bi T (R>L)  R dorsal med parasagittal F  R basal F | Not done | Tight cluster:  R TPO | SEEG, 14 electrodes | R ITG and  R fusiform gyrus | R Basal TO resection including heterotopia | PVNH | NSF  3.5 years  (recurrence at 18 months; Class III) | Bi PVNH | Bi PVNH | P | P |
| P34 | F | R | 30 | 30 | R T | 1. Autonomic -> automotor -> L face clonic  2. Aura (R chest/ upper abdominal pain) | 2-3 per week | IS: R T  SW: R FT | R FT | Subtle hypometabolism  Bi T (R>L)  R F  R FP | R insula  R TPop  22 s | Negative | SEEG, 14 electrodes | R HIPPO/ AMY  R entorhinal  R T pole | R ATL including mesial structures | PVNH | NSF  2.5 years  (recurrence at 22 months; Class III) | Bi PVNH  R AMY enlargement and signal abnormality | Bi PVNH  Additional heterotopia R T horn near AMY | P | P |
| P35 | F | R | 17 | 19 | L PO | Automotor -> Autonomic seizure | 2-4 per month | IRS: L T  SW: L FT | L TP | Subtle Hypometabolism  L mes T  Subtle Hypermetabolism  Ant portion of L O PVNH | Not done | Tight cluster:  L post mid T | SEEG, 14 electrodes | L PO (ant portion of L O PVNH) | L PO (ant portion of L O PVNH) | No tissue (laser) | SF  1.5 years | Bi PVNH | Bi PVNH | C+ | P |
| P36 | F | R | 4 | 20 | R TPO | 1. Aura (somatosensory) -> R face tonic/version -> GTC  2. Aura (somatosensory) -> L face clonic -> L face version -> GTC  3. Aura (vision) | 1-7 per months | IRS: R and L PTO  CS: R PT  SW: R PT  PSPK: R PT | 1. R PT  2. No EEG change | Subtle Hypometabolism  Bi T (R>L)  Bi PO | R dorsal lat P  26 s | Loose clusters:  R post perisylvian  L post central/ supramarginal | SEEG, 13 electrodes | R P PVNH  R POp  R precentral | Laser ablation including R P PVNH and R Pop | No tissue (laser) | SF  1 year | Bi PVNH | Bi PVNH | P | P |
| P37 | M | R | 50 | 56 | L T | Aura (psychic) -> Aphasic -> Complex motor | Daily | IS: Gen, lateralized LH max L T    IRS: L T  PSPK: L T | Nonlocalizable | Subtle Hypometabolism  L basal med TO  Moderate  Hypermetabolism  Bi PVNH | L post lat T  L TP  L basal medial T  R med PO  10 s | Tight cluster:  L post mid T close to PVNH  Loose cluster:  L post perisylvian | SEEG, 14 electrodes | Post portion of L PVNH  L basal temporal including fusiform | Laser ablation of post portion of L PVNH | No tissue (laser) | NSF  1 year  (recurrence within 1 month; Class III) | Bi PVNH | Bi PVNH  Additional PVNH L T horn near HIPPO  Additional PMG L Fop and L supramarginal | P | P |
| P38 | M | L | 20 | 20 | R PO | 1. Aura (nonspecific) -> GTC  2. Dialeptic  3. NCS | Yearly (variable) | IS: R TPO  SPK: R TPO  PSPK: R TPO | R PO | Not done | Not done | Not done | Not done | Not done | Not done | No surgery | NA | R hemimegalencephaly with PMG at R TPO | R hemimegalencephaly with PMG at R TPO | NA | NA |
| P39 | M | L | 10 | 52 | LH | Dialeptic | 1 per 1-2 months | CS: L FT  SW: L FT | 1. Lateralized LH max L CT  2. L FCT | Moderate hypometabolism  Bi T (L >R)  Subtle hypometabolism  L OF  L med F  R P  R FTPop | Not done | Tight cluster:  L supramarginal gyrus / STG  Loose cluster:  L T  Scatter:  L F | Not done | Not done | Not done | No surgery | NA | L peri-sylvian PMG | L peri-sylvian PMG  Additional PMG R post peri-sylvian | NA | NA |
| P40 | M | L | 4 | 36 | Focal, side unclear | Autonomic -> Face Automotor -> Aphasic | 1 per 2 months | SW: Bi FT or L FT | L FT | Negative | L ant med T  L ant insula  L inf F  16 s | Tight clusters:  R ant mes T  L ant mes T | Not done | Not done | Not done | No surgery | NA | R peri-sylvian PMG | R peri-sylvian PMG  Additional PVNH R O and OF | NA | NA |
| P41 | M | R | 23 | 40 | R T | 1. Hypermotor -> L face versive -> GTC  2. Bilateral asymmetric tonic -> GTC | 1-2 per month | CS/IRS: R T    SW: R FT, Bi CTP  SPK: Bi F  PSPK: lateralized LH max L CTP | 1. Lateralized RH  2. Nonlocalizable | Subtle hypometabolism  R T  R TP  R TO | R OF  R ant T  21 s | Scatters:  R lat P  R PO | SEEG, 19 electrodes | R T | R ATL including mesial structures | Negative | SF  1 year | PMG R insula  Closed lip schizencephaly | PMG R insula  Closed lip schizencephaly  Additional PMG L post insula | D | N |
| P42 | F | R | 9 | 24 | L T | Aura (psychic/somatosensory R jaw) -> R face and arm clonic -> GTC | 1-8 times per day | IS: Bi parasagittal, lateralized RH or lateralized LH  SWC: L P or lateralized RH | Lateralized LH max T and CP | Subtle hypometabolism  Bi T (L>R)  Hypermetabolism  L post insula | Bi mes basal F  (R>L)  8 s | Negative | SEEG, 15 electrodes | L Top (posterior border of PMG) | Not done | No surgery | NA | PMG L supramarginal and L posterior sylvian | PMG L supramarginal and L posterior sylvian  Additional PMG R post insula | C+ | NA |
| P43 | F | L | 8 | 19 | LH | 1. R Arm Tonic -> GTC  2. NCS | 1-2 per day | CS: Lateralized LH  IRS: L FT, Bi T (L>R)  SPK: L FC, L T, R PO | 1. L CP  2. LT  3. LF | Not done | L med PO  16 s | Tight clusters:  R PO sulcus  L PO sulcus | Not done | Not done | L hemispherectomy including removal of L HIPPO | HS type II  Glial scar | SF  1 year | L MCA infarct  L HS | L MCA infarct  L HS | NA | P |
| P44 | M | L | 10 | 25 | L P | Aura (somatosensory R arm) -> R arm tonic -> GTC | 1 per month | None | L FC | Subtle hypometabolism  Bi T (L>R)  L F  Bi P  R O | Not done | Not done | SEEG, 14 electrodes | L Pop  L post central gyrus | L P resection | FCD IIId  Glial scar | SF  1.5 years | Bilateral multiple cortical infarct | Bilateral multiple cortical infarct  Additional L mid F infarct | C+ | P |
| P45 | M | R | 17 | 23 | RH | Aura (somatosensory) -> L face tonic -> GTC | 3-6 per month | IRS: R T  IS: L T | R FCT | Moderate hypometabolism  Bi T (R>L)  Subtle hypometabolism  R FTPop  R insula  Bi dorsal F (R>L) | Not done | Tight cluster:  R post perisylvian | Not done | Not done | Not done | No surgery | NA | R F infarct (questionable connection to cortex) | R F infarct (no connection to cortex) | NA | NA |
| P46 | M | R | 24 | 38 | L T | 1. Aura (autonomic) -> Automotor -> Autonomic  2. NCS | 1 per week | IS: L FT, Gen max Bi F  SW: L T | L T | Subtle hypometabolism  Bi T (L>R)  L TP  L TO  R Lat F | Not done | Not done | Not done | Not done | L ATL including mesial structures | HS Type I | SF  2.5 years | R Fop, R T pole and L P infarct    L HS | R Fop, R T pole and L P infarct    L HS | NA | P |
| P47 | M | R | 24 | 25 | MF L&R | 1. Aura (somatosensory, whole body) -> Complex motor  2. Aura (somatosensory, whole body) -> R face tonic -> Axial tonic -> Tonic | 1-2 per day | CS: Gen and lateralized LH  SW: Gen and lateralized LH, max F  SW: L FT  PSPK: Gen max Bi F (L>R) | 1. R FC  2. L F | Not done | L dorsal lat F  L basal F  L Fop  R post insula  22 s | Loose clusters:  R post perisylvian  L post inf T | SEEG, 14 electrodes | L pre SMA, L SFS/MFG, L precentral close to tuber | L F resection including L precentral tuber | Tuberous sclerosis | SF  1 year | Tubers L precentral, L ant T, R P | Tubers L precentral, L ant T, R P  Additional tubers identified Bi F pole | P | P |
